# Postischemic endothelial immune reprogramming in stroke

**DOI:** 10.3389/fimmu.2026.1987918

**Published:** 2026-10-05

**Authors:** Li Gao, Zixuan Liang, Jianqin Xie

**Affiliations:** 1 Admission and Discharge Service Center, The Second Hospital & Clinical Medical School, Lanzhou University, Lanzhou, Gansu, China; 2 Department of Anesthesiology, The Second Hospital & Clinical Medical School, Lanzhou University, Lanzhou, Gansu, China

**Keywords:** blood-brain barrier, endothelial activation, endothelial immune reprogramming, immunothrombosis, ischemia/reperfusion, ischemic stroke, leukocyte trafficking

## Abstract

Large-vessel recanalization is essential in acute ischemic stroke, but it does not guarantee microvascular reperfusion. Endothelial responses may contribute to persistent tissue hypoperfusion through leukocyte trafficking and immunothrombosis. Many of these responses may begin during ischemia and may continue or intensify when blood flow returns. We therefore use postischemic or ischemia/reperfusion-associated terminology unless experiments isolate the contribution of reperfusion. We propose that endothelial immune reprogramming requires a coordinated molecular program, localization to a defined vascular segment and time, and a corresponding change in immune-regulatory function. An isolated increase in an adhesion molecule or circulating biomarker indicates activation or injury but cannot identify such a state. Barrier/stress, adhesive/chemotactic, and prothrombotic responses overlap. Experimental studies implicate endothelial signaling, leukocyte arrest, and platelet interactions with neutrophil extracellular traps, but have not established a common sequence of endothelial states. Human evidence comes mainly from circulating biomarkers, thrombectomy thrombi, post-mortem tissue, and perfusion imaging. The available findings have yet to link a defined cerebral endothelial state to downstream perfusion and clinical outcome in the same patients. Prospective studies should combine serial vascular biomarkers, repeated perfusion imaging, and longitudinal follow-up, with endothelial-enriched molecular profiling when tissue is available. Therapeutic studies should test whether modifying a reversible endothelial program relieves no-reflow while preserving later immune resolution and vascular repair. These approaches remain investigational.

## 1 Introduction

Recanalization restores flow in a previously occluded artery, yet the downstream microcirculation may remain underperfused. In pooled thrombectomy-trial imaging, approximately one quarter of patients with near-complete angiographic reperfusion had tissue-level no-reflow at 24 hours; this pattern was associated with infarct growth, hemorrhagic transformation, and worse functional outcome (1). A subsequent pooled analysis did not detect better functional outcomes in patients with near-complete or complete angiographic recanalization and no-reflow than in those with unsuccessful thrombectomy (2). Residual hypoperfusion is thus associated with clinically important outcomes, but these studies do not establish its endothelial basis or the contribution of reperfusion. Imaging alone cannot determine whether endothelial swelling, leukocyte arrest, platelet-rich microthrombi, pericyte constriction, edema, or distal emboli predominate in an individual patient.

Cerebral endothelial cells (ECs) respond to hypoxia, danger signals, inflammatory mediators, and changes in flow during ischemia and after recanalization. Endothelial cells display chemokines, recruit leukocytes, and regulate immune-cell activity (3). Reviews of stroke describe thrombo-inflammatory injury during both ischemia and reperfusion (4, 5), while the no-reflow literature considers mechanisms within and outside the vessel lumen (6). These processes overlap in time. Here, we examine how coordinated postischemic endothelial responses might connect leukocyte trafficking with downstream immunothrombosis, and where direct evidence is still lacking.

Restoring arterial patency does not necessarily restore tissue perfusion. An endothelium-centered explanation is clinically useful only if it accounts for measurable downstream failure and distinguishes responses to severe infarction from mechanisms that prevent capillary reperfusion.

We use endothelial immune reprogramming as a working term for coordinated changes in endothelial immune-regulatory function over time. The term does not denote an established diagnostic category or imply permanent epigenetic memory. Barrier/stress, adhesive/chemotactic, and prothrombotic responses may overlap; none alone defines a distinct state. Barrier leakage, regulated diapedesis, and microvascular obstruction are considered separately throughout this review, as are the questions of reperfusion dependence and causal involvement in human stroke.

This focused narrative review prioritizes peer-reviewed human ischemic-stroke studies and experimental work relevant to endothelial barrier regulation, leukocyte trafficking, immunothrombosis, and no-reflow. Evidence is grouped by source: Level I, human ischemic-stroke observations; Level II, *in vivo* stroke-model studies, with functional interventions specified where applicable; Level III, *in vitro* or ex vivo mechanistic studies; and Level IV, findings extrapolated from other vascular or central nervous system disorders. These categories do not rank causal certainty or assess risk of bias. Human observations provide clinical context, whereas endothelial-selective experiments can provide more specific mechanistic evidence. We assess whether each pathway has been localized to endothelium, tested functionally, shown to depend on reperfusion, and validated in patients.

## 2 Endothelial responses across ischemia and reperfusion

### 2.1 Definition and relation to reperfusion

Endothelial activation includes stimulus-responsive changes such as adhesion-molecule expression or mediator release, whereas dysfunction denotes impaired vascular function (7). Thrombo-inflammatory activation refers to coupled inflammatory and procoagulant responses (4, 5). Phenotypic switching is a broader term for changes in cellular properties, including immune interactions (3). In this review, immune reprogramming overlaps with these concepts and is not presented as an established separate biological entity. We use it for a proposed endothelial program in which coordinated molecular changes accompany a reproducible change in immune-regulatory function.

Studies in mouse stroke have combined endothelial molecular profiling with vascular localization, temporal sampling, and, in some cases, functional trafficking experiments (8, 9). Drawing on these approaches, we propose three minimum criteria, to be assessed together. First, a coordinated multigene program should be identified in an endothelial-enriched population and supported by protein measurements or another independent assay. Second, the program should be localized to a vascular segment and anatomical region at specified times relative to ischemia and recanalization. Changes within cells must be distinguished from altered subtype abundance or leukocyte contamination. Third, the program should correspond to a measured change in immune-regulatory function in the same or a directly matched vascular context. Relevant functions include leukocyte arrest or passage, chemokine presentation, and platelet/coagulation interactions (3, 4). Barrier leakage alone does not meet this requirement unless it is linked to immune regulation. These are working criteria rather than consensus thresholds. A molecular signature identifies only a candidate state until its function is supported. Establishing a causal role requires endothelial-selective perturbation; persistence beyond the initiating stimulus and permanent epigenetic memory each need direct evidence.

Reperfusion dependence needs to be assessed separately. A response can be considered ischemia-initiated only if its onset is demonstrated before flow is restored. Reperfusion-initiated responses must depend on restored flow for their onset; amplification or maintenance by reperfusion requires an attributable increase in magnitude or duration. Measurements made only after transient middle cerebral artery occlusion (tMCAO) or oxygen-glucose deprivation/reoxygenation generally reflect the combined insult. Serial sampling and flow measurements under matched ischemia-only and ischemia/reperfusion conditions, preferably with intervention at reperfusion, are needed to separate these effects. Permanent and temporary occlusion can produce different infarct volumes and barrier damage at the same post-onset assessment time (10). Their comparison alone therefore cannot isolate reperfusion from differences in ischemic burden and lesion evolution. Reoxygenation of static endothelial monolayers, as used in barrier studies, also lacks the full hemodynamic and circulating-cell context (11). Where the contribution of reperfusion remains unresolved, we use postischemic or ischemia/reperfusion-associated terminology.

### 2.2 Barrier disruption and stress responses

Hypoxia, metabolic failure, and inflammatory signaling can stress the endothelium during ischemia. Reoxygenation and restored shear may amplify selected pathways, but measurements made only after reperfusion cannot establish when they began. Observational studies reported differences in circulating syndecan-1 and other glycocalyx fragments between patients receiving different reperfusion treatments (12, 13). These signals are systemic, and the treatment groups were not randomized. In a mouse photothrombotic stroke model, ischemic tissue showed increased heparanase activity and loss of heparan sulfate; related cell experiments identified acrolein-mediated proheparanase activation (14). This evidence supports postischemic glycocalyx injury without establishing whether it depends on reperfusion or contributes to clinical no-reflow.

Spatial transcriptomics at 24 hours after tMCAO showed reduced claudin-5 (*Cldn5*) expression, altered cytoskeletal-adhesion programs, and increased interleukin-6 (*Il6*) expression in peri-infarct vessels (15). The vascular response is spatially localized, but a single sampling time cannot distinguish ischemic priming from amplification after reperfusion. Endothelial interventions provide evidence of function. Endothelial deletion of the transient receptor potential melastatin 2 gene (*Trpm2*) reduced oxidative calcium overload, plasma leakage, immune-cell infiltration, and injury (16). Endothelial peroxiredoxin-4 loss or gain altered actin stress fibers, junctional redistribution, early leakage, leukocyte infiltration, and long-term recovery (11). In a thromboembolic model and human endothelial cultures, inhibition of lectin-like oxidized low-density lipoprotein receptor 1 and matrix metalloproteinase 9 partly protected the barrier during delayed tissue-type plasminogen activator (tPA) exposure (17). These Level II–III studies show that barrier injury can be modified under the conditions tested. They do not, however, isolate restoration of flow from other model components, including tPA exposure. Reviews of the blood-brain barrier (BBB) describe the relevant pathways (18); leukocyte passage still requires direct measurement.

Barrier dysfunction alone does not establish immune reprogramming. Edema can compress microvessels independently of regulated leukocyte diapedesis, and protection by constitutive gene deletion gives little information about the treatment window after recanalization.

### 2.3 Adhesive and chemotactic responses

Endothelial activation changes how circulating cells engage the vessel wall. In 52 thrombectomy patients, soluble intercellular adhesion molecule 1 (ICAM-1) and vascular cell adhesion molecule 1 (VCAM-1) rose after reperfusion and higher values were associated with worse outcomes (19). Matched comparisons are needed to determine whether these increases reflect restored flow, ongoing ischemic injury, treatment effects, or systemic inflammation. Circulating protein measurements cannot establish a cerebral endothelial source. A 325-patient endovascular thrombectomy (EVT) cohort linked a composite of soluble adhesion and endothelial markers to parenchymal hematoma (20). In human post-mortem tissue, ICAM-1-positive microvessels and granulocytes increased during the first days after stroke, although the series was small and heterogeneous (21). These observations are consistent with endothelial activation or injury. Defining a distinct state would also require coordinated molecular and functional evidence.

Functional models identify pathways that can affect both adhesion and barrier integrity. Endothelial caveolin-1 gain-of-function reduced microthrombosis and myeloid infiltration through a retinoid X receptor γ-related pathway, while its barrier-protective effect was separable from this pathway (22). Deletion of junctional protein associated with coronary artery disease reduced VCAM-1, vascular leakage, and acute injury; the accompanying patient association was prognostic rather than causal (23). Metabolic stress and cell death may also contribute. Hypoxanthine induced calcium-dependent endothelial pyroptosis through gasdermin E in vascular organoids and worsened leakage in mice (24). A multi-model study identified a hyperglycolytic endothelial subset in which lactate-associated histone lactylation and signaling through activating transcription factor 4 and DNA damage-inducible transcript 4 contributed to mitochondrial failure, necroptosis, edema, and inflammation (25). The molecular and functional findings are more informative than an isolated adhesion marker. To identify an immune-regulatory state, the metabolic or cell-death program still needs to be linked to a trafficking function in a defined vascular segment. Its dependence on reperfusion requires separate testing.

Adhesion-molecule expression alone cannot explain leukocyte trafficking. Soluble ectodomains may reflect shedding rather than luminal availability, and whole-brain RNA cannot identify the vascular segment displaying the ligand. To show that an endothelial pathway controls leukocyte entry, studies need spatial localization, a defined leukocyte receptor, altered arrest or transmigration after pathway perturbation, and an outcome assessed at the relevant phase. Few studies meet all of these criteria.

Platelets can also activate the endothelium. In primary mouse brain endothelial cultures, platelet-derived interleukin-1α induced ICAM-1, VCAM-1, and C-X-C motif chemokine ligand 1 and supported neutrophil transmigration (26). This finding supports reciprocal platelet-endothelial signaling in culture, but does not establish reperfusion dependence in stroke. Recruited cells may reinforce a surface already activated during ischemia.

### 2.4 Prothrombotic and antifibrinolytic responses

Endothelial prothrombotic activity can arise during ischemia and persist after recanalization. Endothelial-derived von Willebrand factor (VWF) was the major VWF pool driving fibrin deposition, neutrophil infiltration, and injury in chimeric stroke mice (27). Human thrombectomy thrombi contained substantial VWF, and a disintegrin and metalloproteinase with thrombospondin type 1 motifs 13 (ADAMTS13) lysed experimentally generated tPA-resistant VWF-rich occlusions (28). A constitutively active ADAMTS13 variant reduced VWF/fibrin deposits, platelet aggregates, and neutrophil recruitment after thrombotic MCA occlusion in mice. In a separate tMCAO model with systemic inflammation, treatment 4 hours after reperfusion reduced platelet aggregates and tissue hypoperfusion (29). The interventions support a functional VWF-platelet pathway in ischemia/reperfusion models, but do not establish when endothelial VWF release begins. Effects on large-artery thrombolysis also need to be separated from effects on downstream microvascular no-reflow.

Complement, tissue factor, thrombin, and impaired endogenous anticoagulant functions may amplify thromboinflammation. Deletion of complement component 3a receptor (C3aR) reduced injury after middle cerebral artery occlusion (MCAO); a C3aR antagonist also reduced neutrophil infiltration and barrier disruption, but neither approach isolated endothelial C3aR (30). Platelet inflammatory activity has been described during both ischemia and reperfusion (31), so its onset cannot uniformly be attributed to restored flow. Experimental stroke supports reciprocal signaling among platelets, leukocytes, and endothelium, but the timing and cellular source of individual pathways remain uncertain.

Loss of thrombomodulin, endothelial protein C receptor, prostacyclin, or nitric oxide is mechanistically plausible after oxidative injury, but direct phase-resolved stroke evidence is sparse. Likewise, plasminogen activator inhibitor 1 (PAI-1) expression may create an anti-fibrinolytic surface, yet circulating PAI-1 cannot assign the signal to brain endothelium. Resolving these uncertainties is relevant to treatment because antiplatelet, anticoagulant, and fibrinolytic strategies carry distinct intracranial bleeding risks. Future studies should quantify luminal VWF strings, platelet dwell time, fibrin formation, and capillary flow in the same preparation.

## 3 Endothelial control of leukocyte trafficking

### 3.1 The endothelial-governed trafficking cascade

Leukocyte recruitment begins with selectin-mediated tethering and rolling. Endothelial chemokine display then activates leukocyte integrins. Binding of lymphocyte function-associated antigen 1 or macrophage-1 antigen to ICAM-1, and of very late antigen 4 to VCAM-1, stabilizes arrest. Crawling precedes paracellular or transcellular diapedesis and perivascular positioning. Loss of tight junction signal can facilitate access, but regulated passage differs from bulk barrier leakage. Reviews of immune-cell-mediated BBB injury discuss both their overlap and their differences (32).

Leukocyte passage commonly occurs at post-capillary venules, whereas capillary stalling can reduce flow without transmigration. Intravascular arrest, perivascular retention, and parenchymal entry require distinct readouts. Static adhesion staining, soluble markers, and total infiltrating-cell counts cannot reconstruct this sequence. Intravital microscopy, vascular labeling before dissociation, and endothelial-specific perturbation can distinguish these endpoints across arteries, capillaries, and venules.

Stroke studies implicate the endothelium at several steps of recruitment. Circulating neutrophils and T cells increased after tPA infusion in patients. In an embolic rat model, tPA increased leukocyte adhesion and migration, while modulation of sphingosine-1-phosphate receptor 1 or C-C motif chemokine ligand 2 reduced hemorrhagic transformation (33). These observations do not establish a broadly applicable immune therapy. In mice, a multicomponent intervention reduced P-selectin, ICAM-1, chemokines, leukocyte-endothelial interactions, and no-reflow (34). The treatment affected several pathways, so its benefit cannot be assigned to a single molecular target. The recruitment cascade as a whole warrants further study.

Single-cell profiling has helped describe the timing of these changes. Brain and blood profiling at days 2 and 14 showed that infiltrating leukocytes diverged from circulating states and that endothelial signatures changed early (35). A separate study found ICAM-1-high venous endothelial programs around day 3 and VCAM-1-high programs around day 14, while leukocyte infiltration-related signatures changed less than those at the vascular interface (8). These Level II studies identify candidate endothelial states by relating multigene profiles to vascular identity and sampling time. Functional and temporal comparisons are needed to determine whether the clusters differ in trafficking behavior, represent lineage transitions, or depend on reperfusion.

### 3.2 Cell-type and phase specificity

A 2026 tMCAO study described reactive endothelial venules. ICAM-1 blockade altered acute B-cell entry, whereas later VCAM-1 blockade reduced accumulation of natural killer T cells, regulatory T cells, and natural killer cells and worsened tissue loss and recovery (9). Molecular profiling, vascular localization, and intervention at different phases address several of the proposed criteria for immune reprogramming. Receptor blockade tests only one component, however, and does not selectively perturb the full endothelial state. Because an adhesion pathway may serve different functions over time, these results argue against using anti-adhesion therapy without regard to phase. Reactive endothelial venules and the reported temporal ICAM-1/VCAM-1 pattern have not been validated as a corresponding clinical sequence in patients.

Recruitment also varies by leukocyte subset. Single-cell profiling identified a cluster of differentiation 8 (CD8)-positive T-cell subset expressing C-C motif chemokine receptor 7 (CCR7) in patients with stroke. Mouse experiments implicated endothelial C-C motif chemokine ligand 19 in its recruitment; splenic denervation or CCR7 targeting reduced brain entry and acute injury (36). Endothelial overexpression of atypical chemokine receptor 1 reduced macrophage infiltration and preserved barrier integrity after tMCAO (37). Its efficacy when gene delivery begins after recanalization remains untested. A cascade involving caspase-1, gasdermin D, paxillin, and VCAM-1 linked endothelial pyroptosis to macrophage adhesion in a recent preclinical report (38). Aging also changes the arriving neutrophil pool: altered granulopoiesis generated atypical neutrophils that aggravated mouse stroke (39).

Neutrophils can release proteases, reactive oxygen species, and neutrophil extracellular traps (NETs) within minutes to hours, whereas monocyte differentiation and lymphocyte effects vary across later phases and subsets. Sex, age, diabetes, reperfusion method, and collateral status can alter these kinetics. Serial blood phenotyping needs to be paired with perfusion imaging and endothelial measurements localized to tissue. A fall in circulating cells alone does not establish brain entry.

Recruitment needs to be interpreted in relation to vascular segment, phase, and leukocyte subset. Early neutrophil and monocyte arrest can be injurious, whereas later lymphocyte entry can be damaging, neutral, or reparative. The timing observed in mice still needs confirmation in patients, and transcript enrichment requires functional testing before it can explain trafficking. The same endothelial surface that interacts with leukocytes can support platelet adhesion and extracellular chromatin accumulation. **Table 1** summarizes the overlapping responses, representative functional evidence, limits of attributing effects to reperfusion, and therapeutic hypotheses.

**Table 1:** Endothelial response modules after ischemic stroke.

Endothelial response module	Approx. phase	Endothelial features	Immune or thrombotic partners	Functional consequence	Supporting evidence	Interpretive limits	Therapeutic hypothesis
Barrier/stress	Hyperacute to acute	Glycocalyx loss; junctional and cytoskeletal changes	Neutrophils, macrophages; plasma proteins	Leakage and edema. Immune access requires separate measurement.	Human glycocalyx markers (12, 13). Spatial vascular profiling (15) and endothelial interventions in models (11, 16).	Onset and reperfusion dependence remain unresolved.	Protect glycocalyx and junctions; establish post-recanalization benefit and effects on immune trafficking.
Adhesive/chemotactic	Acute, with later programs	Selectins; ICAM-1; VCAM-1; chemokine display	Leukocytes	Leukocyte arrest and regulated passage are distinct endpoints.	Circulating and tissue activation markers in humans (19, 21). Temporal endothelial programs and functional trafficking data in mice (8, 9).	The human sequence remains unvalidated.	Test segment- and phase-specific modulation while preserving repair-supporting leukocyte entry.
Prothrombotic	Ischemia to early reperfusion	VWF	Platelets, neutrophils, NETs and fibrin	Intraluminal obstruction may contribute to hypoperfusion.	Endothelial VWF and platelet–NET mechanisms in mice (27, 40). VWF and NETs in human occlusive thrombi (28, 41).	Proximal clots do not establish downstream endothelial states.	Test VWF-platelet-NET pathway interruption with flow and hemorrhage endpoints.
Repair/resolution	Subacute to recovery	Barrier recovery; vascular remodeling; angiocrine signaling	Reparative lymphocyte and myeloid subsets	Phase-dependent immune entry	Phase-dependent trafficking (9) and endothelial repair in models (42). Inflammation may impair angiogenesis (43).	No obligatory final state or validated human trajectory.	Preserve reparative trafficking; verify that remodeling restores functional perfusion.

Modules overlap and do not establish distinct states. Phases are approximate and derive mainly from experimental models.

## 4 Endothelium at the interface of inflammation and thrombosis

### 4.1 Platelet-endothelial-coagulation coupling

Platelets adhere to VWF-rich or injured vascular surfaces, release inflammatory mediators, bind leukocytes, and expose procoagulant phospholipids. A study of 45 stroke patients and 45 healthy controls found increased high-mobility group box 1 (HMGB1) on platelet microvesicles and implicated it in NET formation. *In vitro* experiments linked NETs to greater procoagulant activity, while related mouse experiments implicated this pathway in brain injury (44). Another translational study used platelet-specific HMGB1 deletion and a selective NET inhibitor to show that platelets drive acute NET formation without requiring reduced neutrophil recruitment (40). A proadhesive vascular surface could sustain these interactions among platelets, neutrophils, and coagulation factors, but the endothelial contribution is still partly inferred.

Endothelial VWF links inflammation and thrombosis by capturing platelets under shear, while ADAMTS13 limits VWF persistence (27, 29). Platelet P-selectin and CD40 ligand can reinforce leukocyte binding, while platelet phosphatidylserine exposure supports thrombin generation (31). Endothelial-selective experiments need to measure capillary flow and vascular interactions alongside tissue outcome. A smaller infarct alone does not establish relief of microvascular plugging.

Microvascular immunothrombosis occurs downstream of the original large-artery clot and may develop during ischemia, then persist or intensify after recanalization. It is spatially distinct from the occlusive thrombus and may precede reperfusion. Thrombin, fibrin, complement, and platelet-leukocyte aggregates can remain relevant after the proximal artery is open. The immune and hemostatic composition of a thrombus can influence its behavior (5), but a retrieved proximal clot reveals little about the timing or location of downstream endothelial activation.

### 4.2 Neutrophils and NET-amplified endothelial injury

Human thrombectomy thrombi contain neutrophils and citrullinated-histone-positive NETs; ex vivo deoxyribonuclease (DNase) improved tPA-mediated lysis (41). A later analysis of 44 cerebral and 28 coronary thrombi identified thrombus age as an independent predictor of NET content, without a mediating effect of thrombolytic treatment (45). NETs may therefore accumulate before successful recanalization. Retrieved thrombi document NET-associated thrombosis in humans, but cannot characterize downstream capillary endothelium or establish which responses were triggered by reperfusion.

NETs contain extracellular chromatin, histones, neutrophil elastase, and myeloperoxidase. They can promote interactions between platelets and coagulation factors, as well as endothelial injury, which may reinforce inflammation (40, 44). Citrullinated histone H3, myeloperoxidase–DNA complexes, or extracellular DNA alone cannot establish downstream NET burden. Morphological criteria should be combined with flow, endothelial-injury, and coagulation endpoints.

Intervention studies in stroke models support a contribution of NETs to injury. Platelet HMGB1 drove NETs, and neonatal NET-inhibitory factor improved outcomes in aged and diabetic mice (40). Myeloid deletion of pyruvate kinase M2 (PKM2) reduced neutrophil activation, NETs, platelet and fibrinogen deposition, and postischemic hypoperfusion; pharmacological inhibition of PKM2 nuclear translocation also improved outcomes (46). Intra-arterial DNase after recanalization improved flow and outcome in aged mice (47). These interventions support NET-amplified thromboinflammation, but DNase, PKM2 inhibition, and NET blockade have different off-target and infection risks. Broader inflammatory effects, including NET-microglia interactions, are reviewed elsewhere (48). Here, the focus is on NET-mediated endothelial injury, platelet/coagulation interactions, and flow obstruction.

### 4.3 Microvascular plugging and no-reflow

Two-photon imaging after thrombolytic recanalization showed neutrophils stalling in 20–30% of capillaries; anti-Ly6G depletion restored microvascular perfusion and reduced injury (49). A newer multimodal imaging and transcriptomics study also associated neutrophil depletion with improved early microvascular flow and reduced inflammatory programs, with benefit across aged and female mice (50). In the latter study, depletion began before MCAO; its benefit therefore cannot be assumed to extend to treatment after successful recanalization. The experiments implicate neutrophils in postischemic microvascular failure, but do not establish whether reperfusion initiated the obstruction or how much depends on a reprogrammed endothelial state.

The effect of plugging depends on its distribution within the vascular network. Endothelial and astrocytic swelling may narrow the lumen alongside cellular obstruction (6). Assessing no-reflow requires spatial measurements of perfusion; infarct volume or neurological score alone is insufficient. Repeated imaging is needed to distinguish persistent obstruction from delayed reperfusion failure.

Human perfusion imaging detects residual hypoperfusion but cannot resolve individual stalled cells. In experimental studies, neutrophil depletion may also act through mechanisms beyond endothelial signaling. Pericyte constriction, edema, and distal embolization can coexist with immunothrombosis, so endothelial responses are one possible contributor to no-reflow. A direct relationship between a defined cerebral endothelial state, downstream perfusion, and outcome has yet to be shown in the same patients. **Figure 1** summarizes the timing of these responses, the evidence needed to define an endothelial state, and the work still needed for human validation.

**Figure 1:**
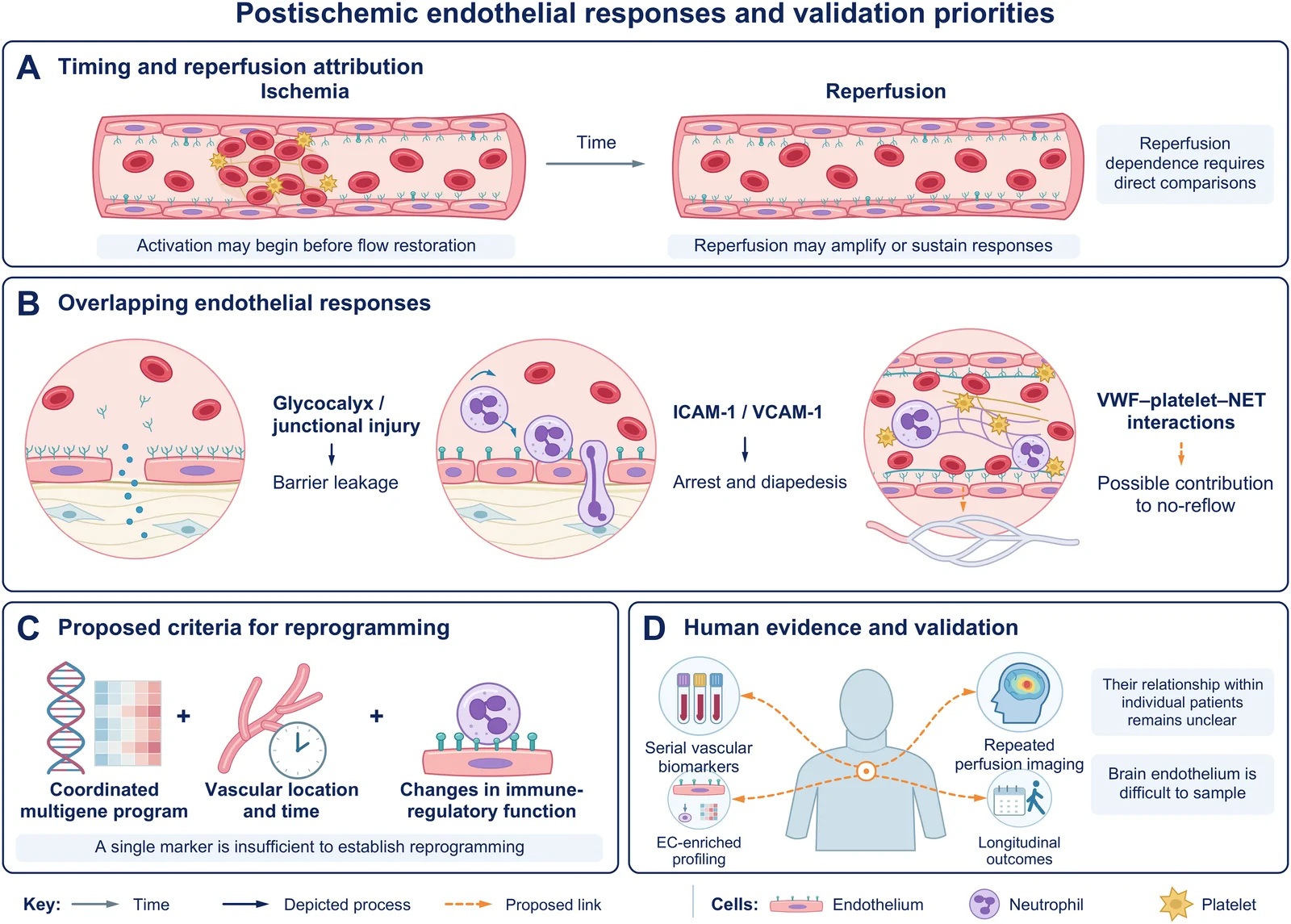
Postischemic endothelial responses and validation priorities. **(A)** Responses may begin during ischemia and increase or persist after reperfusion. **(B)** Barrier injury, leukocyte recruitment, and immunothrombosis overlap. Whether they represent distinct endothelial states remains uncertain; no-reflow has multiple causes. **(C)** Proposed criteria include coordinated molecular changes, vascular location and timing, and immune-regulatory function. A causal role requires endothelial-selective perturbation. **(D)** Human studies should relate endothelial profiles to serial biomarkers, perfusion, and outcomes in the same patients when tissue is available. Gray arrows indicate the passage of time, navy arrows show the processes depicted, and orange dashed arrows indicate proposed links. The strength of evidence differs among these processes. Diagrams are schematic.

## 5 Translation and future directions

### 5.1 Human evidence and its limitations

Human evidence comes mainly from circulating biomarkers, thrombectomy specimens, post-mortem tissue, and perfusion imaging. None captures the proposed mechanism in full. Glycocalyx fragments indicate vascular injury without identifying a cerebral endothelial source (12, 13). Soluble adhesion molecules likewise reflect systemic activation (19, 20). Retrieved thrombi contain evidence of VWF- and NET-associated thrombosis in the occlusive clot, but do not characterize downstream capillary endothelium (28, 41). Post-mortem tissue permits anatomical localization but captures a single time point, overrepresents fatal cases, and may lack complete treatment and recanalization information (21). Perfusion imaging relates residual hypoperfusion to outcome without identifying the responsible cell or molecular program (1, 2). The studies reviewed here have yet to connect a defined cerebral endothelial state, downstream microvascular failure, and disability within the same patients.

Experimental models allow cell-selective perturbation and repeated vascular imaging, but clinical translation requires attention to age, sex, and comorbidity (51). Collateral circulation and species-specific immune biology also need consideration (3, 6). Filament withdrawal, thrombolysis, and thrombectomy differ in how they restore flow; tPA exposure can itself modify barrier injury (4, 17). Protection with constitutive gene deletion, viral delivery before injury, or prophylactic depletion cannot by itself demonstrate reversal of established no-reflow (11, 50). Methodological work in mouse kidney shows that tissue dissociation can alter endothelial recovery and transcription, a technical concern that also needs testing in stroke samples (52). Sampling region is another consideration when interpreting spatially heterogeneous vascular responses (15). Candidate states need confirmation across models and in human tissue; their timing in mice cannot directly define treatment windows in patients.

### 5.2 Therapeutic hypotheses and phase specificity

Therapeutic studies should specify the vascular function they target and the timing of treatment. Possible approaches around recanalization include preserving the glycocalyx and junctions, modifying harmful adhesion or chemokine display, and interrupting VWF-platelet-NET-complement signaling. Endothelial caveolin-1 and peroxiredoxin-4 have been tested in mouse stroke models (11, 22). Other experiments have targeted the VWF pathway with ADAMTS13 variants or NET formation with inhibitors (29, 40). C3aR antagonism has also reduced injury in mice (30). Clinical efficacy remains unknown. These results neither identify a shared endothelial state nor establish that reperfusion initiated the targeted pathway.

The same intervention may have different effects at different stages. Broad anti-adhesion or anti-inflammatory treatment may affect multiple leukocyte subsets, infection susceptibility, and recovery as well as acute injury. The effects of late VCAM-1 blockade suggest that an adhesion pathway targeted for acute inhibition may later support lymphocyte entry during repair (9). A circular RNA intervention promoted peri-infarct vascular repair in mouse and monkey photothrombotic models; mechanistic experiments implicated endothelial RNA regulation by fat mass and obesity-associated protein (42). A separate mouse study linked pathological α-synuclein to acute endothelial inflammation and impaired subacute angiogenesis (43). Post-stroke angiogenesis is related to these responses but remains a distinct endpoint: vessel growth does not necessarily mean restored perfusion or functional repair (53).

### 5.3 Validation in patients

A prospective cohort of patients with successful recanalization could combine serial blood sampling, perfusion imaging, and follow-up, all timed to stroke onset and confirmed restoration of flow. One possible sampling schedule is before EVT where feasible, immediately after recanalization, at 6–24 and 48–72 hours, and during early recovery; this schedule would need prospective testing. Candidate assays include soluble ICAM-1/VCAM-1 and glycocalyx fragments, which have been measured in stroke cohorts (12, 19). VWF, NET markers, and platelet-leukocyte aggregates could be added as exploratory measures of thromboinflammation (31, 44). Complement products could also be explored, given the experimental evidence for complement signaling (30). These systemic markers have not been validated for classifying cerebral endothelial states. Building on clinical no-reflow imaging studies (1, 2), baseline and repeat computed tomography or magnetic resonance perfusion imaging would require prespecified timing and definitions, with assessment of proximal patency, reocclusion, residual distal occlusion, infarct extent, and collaterals. Biomarker trajectories could then be related to persistent or delayed hypoperfusion and subsequent outcomes in the same patients.

Experimental stroke studies provide precedents for endothelial single-cell profiling and spatial analysis (8, 15). We propose adapting these approaches, with single-nucleus or protein assays where appropriate, to clinically obtained surgical or post-mortem tissue. Molecular findings should be paired with clinical measurements whenever possible, with documentation of the vascular region and treatment history. Serial sampling of living human brain microvascular endothelium is rarely feasible, so parallel tissue cohorts and experimental models will often be needed. They can help test whether candidate programs are conserved, but cannot replace validation within individual patients. Analyses should confirm endothelial identity, vascular segment, cell composition, and transcript-to-protein agreement. Neither thrombectomy thrombi nor circulating endothelial-associated signals necessarily represent downstream capillary endothelium.

Follow-up should assess early neurological change, infarct growth, hemorrhagic transformation, and functional outcome, for example the modified Rankin Scale at 90 days (1, 2). Evidence of injury, target engagement, state classification, and prognostic value should be evaluated separately. Analyses need to account for baseline severity, ischemic duration, collaterals, infarct burden, reperfusion grade, treatment, comorbidity, and infection. A consistent temporal sequence and independent replication would strengthen the findings; statistical mediation alone cannot establish causality (54). To be clinically useful, a state should add information beyond these factors and correspond to an experimentally modifiable vascular function.

Preclinical studies should combine endothelial-selective perturbation with simultaneous measurements of leukocyte trafficking and capillary flow. Testing reperfusion-specific hypotheses requires time-matched ischemia-only and ischemia/reperfusion groups, subject to the limitations discussed in Section 2.1. Delayed treatment after confirmed recanalization should be tested in aged, hypertensive, and diabetic animals of both sexes (51). Reviews of acute-stroke thromboinflammation describe candidate targets (55). Before imaging-defined no-reflow or a molecular state can guide trial enrollment, the measurement and a pharmacodynamic response need to be validated. Studies should assess hemorrhage, infection, coagulation, and later functional recovery. These approaches remain research proposals and are not ready for clinical use.

## 6 Conclusion

Postischemic endothelial responses may connect leukocyte trafficking and immunothrombosis with microvascular failure. Responses that begin during ischemia must be distinguished from those amplified or sustained by reperfusion. We propose reserving immune reprogramming for changes supported by coordinated molecular, spatial-temporal, and functional evidence; isolated activation markers or barrier injury are insufficient. Experimental studies support individual pathways and candidate states, but their counterparts in human cerebral endothelium and their contribution to no-reflow remain incompletely established.

Further work needs to establish whether a defined endothelial program predicts persistent hypoperfusion after successful recanalization and contributes to it functionally. This requires serial biomarkers, perfusion imaging, endothelial-enriched profiling where feasible, and longitudinal clinical outcomes, alongside endothelial-selective experiments. Any treatment must preserve the benefits of reperfusion and allow later reparative immune trafficking and vascular remodeling.
